# Applying Adult Learning Theories in Improving Medical Education in Nepal: View of Medical Students

**DOI:** 10.31729/jnma.5292

**Published:** 2021-02-28

**Authors:** Prathana Subedi, Mahendra Pandey

**Affiliations:** 1 Nepalese Army Institute of Health Sciences, Sanobharyang, Kathmandu, Nepal; 2 National Medical College and Teaching Hospital, Birgunj, Nepal

Entering into medical school from practicing or experiencing pedagogic teaching in high school and undergoing a huge transformative process during the 5 years to come out as adults requires self-reflection on what university or medical school has taught us. Adulthood is inevitable in medical school and thus, a question remains, would andragogic teaching be better for me? As an adult medical student, do I learn in a better way than attending the lectures online or in the lecture hall itself? These questions have randomly crossed our minds and the COVID-19 pandemic has made it even more significant.

Andragogy took its shape during the 1980s when Malcolm Knowles further elaborated the concept of andragogy.^1^ It states that adults are:

- Independent and self-directing
- Have diverse experiences
- Integrate learning as a demand of their everyday life
- Are more interested in problem-centered approaches
- Are motivated by internal rather than external drives

Lockdown and self-reflection have made a whole new paradigm shift on what we thought learning was. We generally have different experiences in our lives based on the region of the world where we live, but the “fear of COVID” has been an experience shared by people all around the world. It made humanity unite and understand what everyone was going through. Many webinars, open-access lectures, talks and symposium made realization dawn that we can learn on our own, the things we feel there is a need to learn. The world suddenly opened up for us, and learning happened in a way we wanted to learn, besides the university's online lectures. This is what the personal definition of andragogy was for us.

## APPLYING ADULT LEARNING THEORIES INTO OUR MEDICAL EDUCATION OPTIMIZING STUDENT INVOLVEMENT IN DECISION MAKING AND PLANNING

AMEE (Association for Medical Education in Europe) advocates students' engagement in management, provision of academic programs and their involvement in the local and academic community, and a medical curriculum for the students and by the students. The optimal model is to achieve student and teacher equity in learning. We still have a long way to ensure student involvement in medical education but paving the way is paramount to uplift the health care system of Nepal.

## SELF DIRECTED LEARNING (SDL)

SDL (Self-directed learning) has been the core of adult learning. A study done in a medical college in Nepal, which judged undergraduate students' readiness to take up SDL (Self-directed learning), showed that 72.7% were ready to incorporate self-directed learning in their education.^2^ This has a personal implication for us too. During the lockdown, as a regular program for Walter E. Dandy Neuro Club of Nepal, we had set up a simulation session for students of 1^st^ and 2^nd^ year of medical school to conduct a program on “ArterioVenous Malformation.” The neurosurgery topic seems vast and complex because it being a super specialization. However, the students presented it wonderfully with all the evidence-based approaches, surgical techniques, and the recent trials being conducted on this topic. This made us self-reflect on how much of learning we were doing in medical school and how capable we are of learning independently.

## PROBLEM BASED LEARNING

“Adult learning is self-directed and interested in problem-centered approaches with the application of their learning to solve immediate problems.” The application of Problem-based learning in our curriculum was adapted from the McMaster University of Canada in 1960.^3^ Problem-based learning is a teaching method that applies the same principles of andragogy. A study done in CMC (Chitwan Medical College) found that 78% of students recommend it as being better than the lectures.^4^ It is a learning method based on the principle of using problems as a starting point for acquiring and integrating new knowledge. It is a way of dealing with a problem by integrating diverse aspects of a case, setting learning goals and coming up with a solution; this encompasses all matters of adult learning. It makes the learning environment more conducive and humane and employs a very important self-reflection tool for learners. Subjectively speaking, the Problem Based Learning that we had in the 2^nd^ year of medical school in NAIHS was an effective way of learning to find out answers for ourselves. Actively involving in the learning process made learning easier, applicable and self-paced.

## SKILL-BASED TEACHING

Skills are indispensable for medical students. The actual response is very different from knowing the theory. Skills are not focused enough for medical students in Nepal and are set aside for internship, a yearlong posting in the hospital when we have actually formally completed our medical education. Imparting skills like venepuncture, cannula insertion, delivering a baby, putting on an endotracheal tube, performing CPR, and taking a nasopharyngeal swab could be imparted to medical students from the curriculum itself by employing various techniques like using mannequins and cadavers. The possibilities are endless.

## BUILDING COMMUNICATION SKILLS BY SIMULATION

Medical students need to be taught skills and professionalism; this ultimately amounts to improved patient outcomes and better healthcare systems for Nepal. “Medical paternalism'' Doctor knows best is changing with the increased economic growth in the region.^5^ It has been shown in a study that medical students' empathy levels plummet from the time they entered medical school.^6^ Amongst all this, the use of simulation to teach medical students communication skills and counseling is of utmost importance. Many diverse topics could be learned in smaller groups like breaking bad news, empathy, doctor-patient relationship. This helps gear students towards familiarity when encountering such situations and makes learning active and fun via role-plays and discussion.

## FUN TRIVIA AND QUIZZES

Quizzes use the active recall technique, which serves to enhance retention. Many online software that came to light during this lockdown; like Menti and Kahoot, make medicine something to be learned while having fun and their interface also provides a place to connect with a global audience and, in this case, medical students from many different countries.

## WAY FORWARD

Adult-based learning can have a lot of implications: for it is a concept that empowers adults to study on a priority basis, which they decide, based on their learning plan, their experiences, immediate need, or interest. This certainly improves outcomes. A lot of experience sharing, practical discussion and horizontal transfer of knowledge with collaboration is the ultimate way to learn because learning is and should be fun and impactful. The burden lies on us students too. In a rapidly evolving global era, we need to evolve. We need to feel we are a part of adult learning and start paving a way to achieve student-teacher equity and develop a concept of medical curriculum for the students.
